# Optimization of Polydopamine Coatings onto Poly–ε–Caprolactone Electrospun Fibers for the Fabrication of Bio-Electroconductive Interfaces

**DOI:** 10.3390/jfb11010019

**Published:** 2020-03-17

**Authors:** Simona Zuppolini, Iriczalli Cruz-Maya, Vincenzo Guarino, Anna Borriello

**Affiliations:** Institute for Polymers, Composites and Biomaterials (IPCB)—National Research Council of Italy, V.le Kennedy 54, 80125 Naples, Italy; simona.zuppolini@cnr.it (S.Z.); cdiriczalli@gmail.com (I.C.-M.)

**Keywords:** polydopamine, electrospun fibers, polycaprolactone, electrical conductivity, in vitro response

## Abstract

In recent years, mussel adhesive proteins have attracted much attention because they can form strong adhesive interface interactions with various substrates in a wet environment. Inspired by their catechol- and amine-based molecular structure, polydopamine (PDA), a dopamine derived synthetic eumelanin polymer, was recognized as a suitable bio-interface coating. PDA was successfully used to improve adhesion due to the availability of copious functional groups for covalently immobilizing biomolecules and anchoring reactive species and ions. Recently, it has been demonstrated that PDA and its derivatives can be successfully used for the surface modification of implants interfaces to modulate in vitro cellular responses in order to enhance the in vivo functionality of biomedical implants (i.e., prosthesis). Herein, we propose the development of multifunctional scaffolds based on polyε–caprolactone (PCL) electrospun fibers coated with PDA via electro fluid dynamic methods, by optimizing polymerization/oxidation reactions capable of driving PDA self–assembly, and, ultimately, investigating the effects on cell response. Morphological analyses have confirmed the possibility to obtain different surface topographies as a function of the coating process while in vitro studies proved the ability of PDA coating to interact with cells no compromising in vitro viability. In perspective, in vitro conductive properties of fibers will be further investigated in order to validate their promising use as bioconductive interfaces for tissue engineering applications.

## 1. Introduction

The design and fabrication of biocompatible materials achieving specific performances in biomedical applications still represents a significant issue. Among diverse 3D structures, fibrous scaffolds are considered advantageous for the unique properties at micro and submicrometric scale (i.e., high surface area/volume ratio, reduced fiber diameter, high porosity and light weight) [1]. Their peculiar architecture is able to mimic extracellular matrix (ECM) properties influencing cell attachment and consequently being useful in functional systems as scaffolds for cell culture, tissue engineering and medical textiles [2].

Electrospinning is one of the most promising technologies to produce micro and nanofibers from a large variety of synthetic or natural polymers by using an electrically driven jet of polymer solution or melt [3]. Moreover, scaling of fiber diameter, chemical and morphological features able to address different cell behaviors can be provided by tuning a large number of variables relating to polymer solution properties and process parameters [4]. Among biomaterials used for electrospun fibers production, poly–ε–caprolactone (PCL) is one of the most biodegradable and biocompatible polymers, which has received significant attention because of the environmentally friendly response, thus making it suitable for biomedical applications [5]. The great interest in PCL as a biomaterial in the tissue engineering applications area is largely due to the following advantages: it is non–toxic, it has a slow degradation rate, and the products of biodegradation are non–toxic [6]. However, being a semicrystalline hydrophobic polymer, PCL shows poor cell adhesion and stimulation of cell activities. This limitation can be overcome by providing hydrophilic groups to enhance both cell adhesive properties and creating an environment favorable for proliferation and cell interactions [2]. In this context, several strategies based on surface treatments or blending through the use of bioinspired materials have been exploring how to improve the hydrophilicity and so the biocompatibility of PCL electrospun fibers, without compromising material properties [7,8]. Among the approaches based on surface modification, the use of mussel-inspired polydopamine (PDA) is emerging as really promising, due to its additional attractive properties including strong adhesiveness on moist surfaces, availability of functional groups for immobilization of bioactive molecules and its amenability to post-functionalization via catechol chemistry [9]. PDA coating is considered an advantageous bio-interface also in terms of simplicity, inexpensiveness and low cytotoxicity. The PDA ability of modulating specific in vitro cellular responses is due, not only to its hydrophilicity, but also to the electrical properties provided by the π–electron system [10]. 

As is well known, PDA deposition onto a polymeric substrate occurs during the self-polymerization of dopamine in alkaline environment and in presence of oxygen [11]. Hence, a current challenge is to optimize the synthesis of PDA to improve the homogeneity of the deposition, in order to really generate effective and functional interfaces with cells. In this context, recent development of processing techniques based on a controlled deposition of polymer solutions assisted by the application of high voltage electric fields may offer a promising route to improve the coating onto the fiber surface [12]. 

In this article, we report about the development of PCL fibrous platforms coated with PDA by electrofluidodynamic (EFD) deposition techniques. In particular, PCL electrospun fibers were fabricated, preliminary hydrophilyzed by NaOH treatment and then, functionalized by PDA coating, obtained by spontaneous oxidative self–polymerization of dopamine precursor deposited on fibers by EFD technologies. The optimization of the PDA coating process was designed. Surface morphology and thermal behavior were analyzed on treated and untreated PCL fibers. Biocompatibility of PDA-coated PCL fibers were examined by comparing results with those of untreated ones.

## 2. Results and Discussion

In recent years, several studies have demonstrated the suitability of PCL electrospun fibers as scaffolds for tissue engineering, due to their ability to promote in vitro interactions with cells [13]. Although electrospun PCL mimics many aspects of natural ECM structure, its poor hydrophilicity leads to inadequate cell adhesion, proliferation and differentiation. A surface modification strategy was adopted to introduce suitable functionalities on the PCL electrospun fibers to enhance its hydrophilicity and biocompatibility properties.

**Figure 1:**
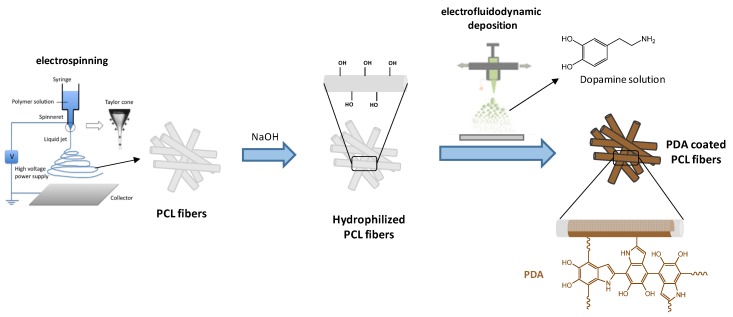
Scheme of the production process: polyε–caprolactone (PCL) fibers were fabricated by the electrospinning and treated in NaOH solution. Polydopamine PDA coating was successively deposited by electrospray of dopamine precursor solution.

The entire process involves PCL fibers fabrication by electrospinning, alkaline treatment by sub–immersion of fibers and PDA coating by oxidative self–polymerization of dopamine under basic conditions. The preparation of the fibers has been efficaciously schematized in Figure 1. 

A simple method to coat a fiber surface with PDA is the immersion into a tris–buffer solution (pH 8.5) of dopamine, which self–polymerizes in the presence of air oxygen as oxidant. Herein, the dopamine solution was electrofluidodynamically deposited until to obtain a thin functional coating. It is noteworthy that PDA was not only deposited onto surfaces, but also formed as dark-black precipitates (particles/aggregates) in suspension due to its insolubility. Based on this assumption, EFD deposition of dopamine solution on PCL fibers was started immediately after dopamine dissolution (T_0_) to ensure the presence of unreacted precursor on the substrate and an anchoring at the early stage of PDA formation. Thus, the oxidative polymeryzation and progressive PDA formation can be carry on both during and after the EFD process until 45 min. PDA coating formation was confirmed by the gradual color change of the surface substrate to dark brown within 6–12 h. 

The hydrophilic properties of electrospun fibers were investigated by wettability measurements. The water contact angle of PCL electrospun fibers was measured to be 115.0 ± 1.0°, confirming their hydrophobic nature. After NaOH treatment, a high decrease in the water contact angle to 35° ± 1.0° was observed due to a significant increment of surface hydrophilicity, thus indicating an evident switch of the PCL fiber properties from hydrophobic to hydrophilic one. Interesting results were obtained in the case of PDA coating NaOH treated PCL fibers: during the experiments, the water drop was immediately absorbed into fibrous networks, resulting in a null contact angle. This further decrease in the water contact angle indicates, confirmed that PDA coating do not negatively influence the hydrophilic properties of the fiber surface, mainly attributed to the alkaline treatment and also corroborates the hydrophilic effect by influencing locally the surface roughness, in agreement with previous studies [14].

**Figure 2:**
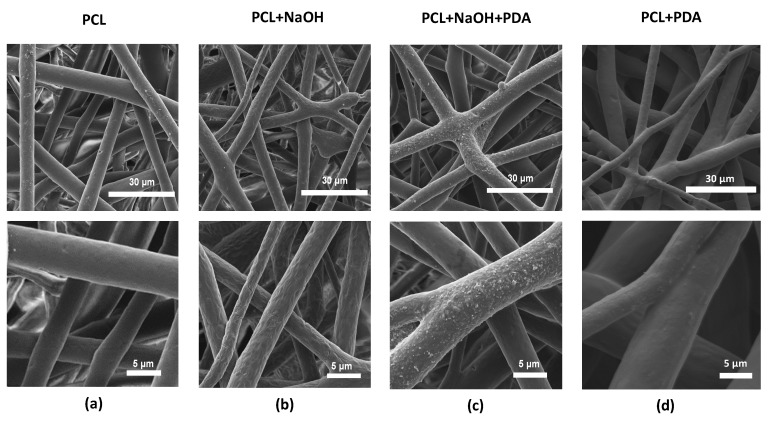
Scanning electron microscope (SEM) images of (**a**) PCL electrospun fibers, (**b**) NaOH-treated, (**c**) PDA-coated NaOH-treated PCL fibers and (**d**) PDA-coated untreated PCL fibers.

These morphological effects of chemical post–treatments on PCL fibers were observed by SEM images (Figure 2). After the alkaline treatment, it was remarked the presence of small islets of PDA onto the surface, originated by the chemical interaction with the NaOH treated surfaces (Figure 2c) that basically contributes to influence topographic features (i.e., roughness), (Figure 2b). Accordingly, in the case of untreated PCL fibers, no evidence of PDA islets was reported (Figure 2d). In overall, comparable size and fiber distributions can be recognized by SEM images, independently upon the applied treatments. 

**Figure 3:**
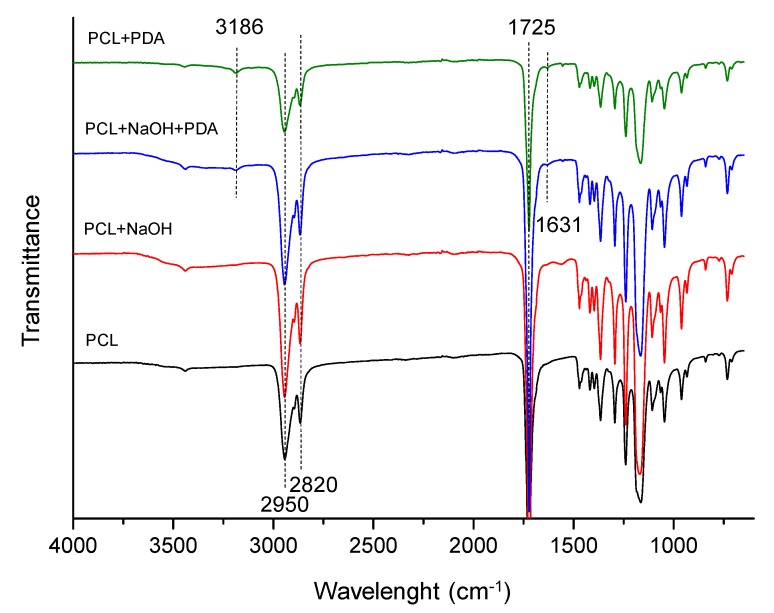
ATR–IR spectra of PCL electrospun fibers: untreated (black curve), NaOH-treated (red curve) and PDA-coated (blue curve), and PDA-coated untreated PCL fibers (green curve).

Attenuated Total Reflectance–Fourier Transform Infrared (ATR–FTIR) spectra of untreated and differently functionalized PCL electrospun fibers were reported in Figure 3. In the case of untreated fibers, PCL characteristic peaks at 2950 and 2820 cm^−1^ are attributable to ν(C–H) stretching, while ν(C=O) stretching was observed at 1725 cm^−1^. After NaOH treatment, the characteristic peaks of PCL fibers were relatively well–retained, with a broadband in the 3600–3200 cm^−1^ range associated to –OH groups confirming that hydrophilicity was induced. Similarly, also after PDA coating, the spectrum is similar to the previous one. Key absorption of cathecol groups, and ν(O–H), are included in the 3600–3100 cm^−1^ range while new weak peaks at 3186 cm^−1^ and 1631 cm^−1^ are associated to ν(N–H) of free amines and the stratching of aromatic C=C bonds in indole, respectively. These latter peaks and the signal at 3186 cm^−1^ were equally observed in the PCL+PDA spectrum where, however, no broadband is clearly observable in the 3600–3200 cm^−1^ range suggesting a lower content of –OH groups. 

**Figure 4:**
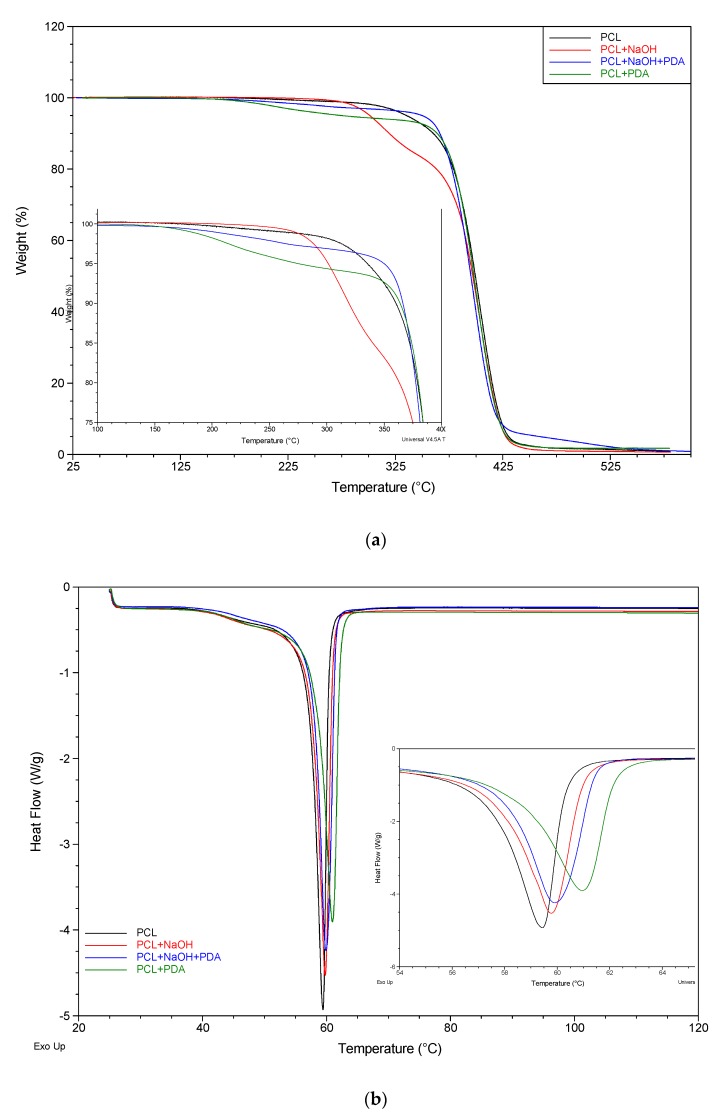
(**a**) TGA and DSC (**b**) curves of PCL fibers: untreated (black curve), NaOH treated (red curve) and post PDA coating (blue curve).

In order to evaluate the contribution of PDA on the structural properties of the fibers, thermal analysis was assessed (Figure 4) and data were summarized in Table 1. Thermogravimetric analysis (TGA) was used to characterize the thermal stability of untreated and treated PCL fibers, and the decomposition in N_2_ atmosphere is shown in Figure 4a. In all the reported curves a similar thermogram profile can be observed with relevant differences detectable as a function of specific treatment. The untreated PCL fibers (black curve) shows a good thermal stability with a thermal decomposition temperature T_max_ of 403 °C. The NaOH treatment (red curve) does not affect the thermal stability of PCL fibers with a T_max_ decrease of only 0.7 °C. However, alkaline pretreatment on PCL fibers seems to have a crucial role in the PDA coating deposition. In particular, a higher thermal stability was observed after PDA coating (blue curve) if compared to PDA coated untreated PCL (green curve) in the first stage of decomposition (120–325 °C). Furthermore, a decreased weight loss of NaOH+PDA-treated PCL fibers in the 425–600 °C range indicated the deposition of PDA layer attributed to decomposition PDA [15]. These results could be attributed to the high affinity of –OH groups provided by NaOH on PCL surface with amine and catechol moieties of PDA which consequently shows a stronger surface adhesion. Figure 4b shows a comparative analysis of differential scanning calorimeter (DSC) thermogram curves from PCL electrospun fibers after different treatments. The melting heat of PCL fibers (black curve) is identified by the endothermic peak at 59.4 °C. For differently treated PCL fibers very slight shifts in Tm were observed. This result is comprehensible considering that hydrophilicity modification was performed at surface level and PDA coating consists of a thin layer.

It is reasonably assumed that the alkaline pretreatment on PCL fibers might ensure a homogeneous hydrophilicity suitable to address both the self–assembly and attachment of PDA on PCL fiber surface. In this context, the morphology of the PCL fibrous scaffold can be controlled, and, consequently, cell interaction mediated with the surrounding materials. Based on these considerations, we focused our investigation on PDA-coated samples obtained from alkaline-treated PCL fibers. 

**Figure 5:**
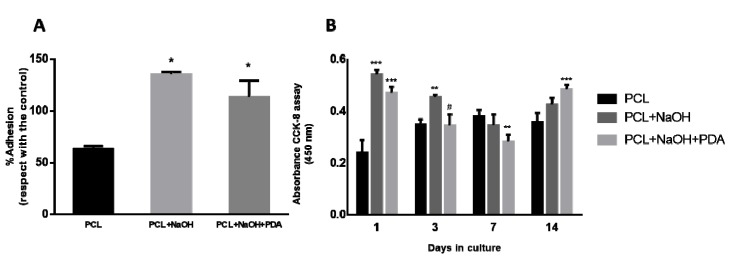
(**A**) Cell adhesion of hMSC onto treated PCL fibers (* significant difference against untreated PCL fiber; *p* < 0.05). (**B**) Cell viability of hMSCs with CCK–8 assay. (* refers to significant difference against untreated PCL fibers; # refers to significant difference against NaOH treated PCL fibers).

PDA-coated electrospun fibers were investigated from biological point of view. First of all, the viability of the adhesion of human mesenchymal stem cells (hMSCs) was evaluated in all groups by Toluidine Blue. An evident increase of signal is associated to the use of surface treatments (i.e., NaOH and PDA) able to significantly increase the hydrophilic properties of the PCL surface (Figure 5A). Results showed that all the treatments promoted the cell adhesion with respect to nude PCL, at 24 h, concurring to support the initial cell–material interaction and the subsequently cellular behavior. No significant differences in hMSC response were detected among the different surface treatments.

Viability tests performed via cell counting kit-8 (CCK–8) confirmed a significant growth of hMSC in culture until 14 days. The presence of PDA did not induce cytotoxic effects on hMSC, but statistically significant differences in terms of viability were recognized in comparison with untreated PCL fibers. At the beginning times, the hydrophilic properties of the fibers – due to the PDA coating and/or the alkaline treatment effects – concur to drastically increase the viability respect to untreated PCL fibers (*p* < 0.001). Contrariwise, at the day 7, a decrease in cell proliferation was detected in the case of PDA-coated samples, probably due to the internalization of PDA fragments released from the coating—in agreement with recent reports on the PDA toxicity on healthy and tumor cells [16]. However, at day 14, this effect was fully balanced by the biocompatible response of PDA coating as confirmed by the increase in cell proliferation respect to untreated fibers (*p* < 0.001).

## 3. Materials and Methods 

### 3.1. Materials 

Poly–ε–caprolactone (PCL, M_w_ 45 kDa) in pellet form (33% w/v), chloroform, sodium hydroxide (NaOH), dopamine hydrochloride (DA), ethanol, 2–amino–2–(hydroxymethyl)–1,3–propanediol (Tris base) and phosphate buffered solution (PBS) were provided by Sigma Aldrich (Milano, Italy). All chemicals were of analytical grade and used as received.

### 3.2. Electrospun Fibers Fabrication

Electrospun fibers were fabricated by using commercialized electrospinning equipment (NF500 – MECC, Fukuoka, Japan) in agreement with the preparation reported in previous studies [17]. Briefly, the PCL solution was processed by selecting 16 kV as the voltage, 0.5 mL/h as the flow rate and 150 mm as electrode distance. Final samples were obtained after a deposition time of 30 min, under environmental conditions (T = 25 °C) and controlled humidity degree (RH = 45%). Fiber surface was also treated in NaOH solution (5 M) at room temperature, for 24 h in order to improve the hydrophilicity. In this case, specimens were removed from the water bath and washed again several times with distilled water until the supernatant pH reached 6.4 and then, dried for 1 h under the hood before the next treatment.

### 3.3. PDA Coating onto PCL Fibers 

PDA coating was performed by a drop-to-drop deposition of a Tris base 10 mM (pH 8.5) solution containing DA (2 mg/mL) onto NaOH–treated PCL fibers by the support of Electro fluid dynamic techniques. Deposition was performed immediately after dopamine solution. The deposition parameters were 22 kV as applied voltage, 2 mL/h as feed rate, at 220 mm of distance with a transverse movement of needle at 3 mm/s. 

### 3.4. Characterization of PCL Electrospun Fibers

The morphology of samples was investigated by images recorded by scanning electron microscopy (SEM, Quanta FEG 200 FEI, Eindhoven, The Netherlands) working at low voltage electron emission (2 kV), with the support of image analysis for fiber size quantification. 

Hydrophilicity of electrospun fibers was evaluated in terms of contact angle measurements by using a water contact angle system (WCA) supported by videocam equipment OCA20 (Dataphysics, Bergamo, Italy). Five measurements with a single droplet (volume, 5 µL) were used for each test. All measurements were performed at time zero to eliminate any influence of subsequent perfusion flow through the membrane. Contact angle size was reported as mean ± standard deviation.

Attenuated Total Reflectance–Fourier Transform Infrared (ATR–FTIR) spectra were recorded on a Perkin Elmer Spectrum 100 FTIR spectrophotometer (Milano, Italy) in the 4000–400 cm^−1^ region. 

Thermal stability of samples was investigated by thermogravimetrical analysis (TGA) by using a Q500 system by TA Instruments (New Castle, Germany) under N_2_ atmosphere (50 mL/min) and a heating ramp of 10 °C/min. Sample weights of around 6 ± 0.5 mg were used for the run test performed by heating from ambient temperature to 600 °C.

Thermal behavior of samples was investigated by differential scanning calorimeter (DSC) tests were performed by a Q1000 DSC (TA Instruments, New Castle, Germany)) under nitrogen atmosphere (50 mL/min) and a heating ramp of 10 °C/min.

### 3.5. Biocompatibility Studies 

Biological assays were performed by using human mesenchymal stem cells (hMSC), which were cultured in Eagle’s alpha minimum essential medium (α–MEM) supplemented with 10% fetal bovine serum, antibiotic solution (100 µg/mL streptomycin and 100 U/mL) and l–glutamine (2 mM), incubated at 37 °C in humidified atmosphere with 5% CO_2_ and 95% air. 

The hMSCs were seeded at 1 × 10^5^ onto PCL- and PDA-coated fibers for adhesion in standard cell culture, for 24 h. After those time periods, samples were rinsed three times with phosphate buffered solution (PBS) to remove the unattached cells. Then, the adhered cells were fixed with 4% paraformaldehyde (PFA) and incubated with 0.1% toluidine blue for 3 h. After incubation time, stain was retired and samples were washed several times to remove the excess of dye. The dye was extracted with 0.1% of sodium dodecyl sulfate and the optical absorption was quantified by spectrophotometry at 600nm. The tissue culture plate was taken as a control. 

As for cell viability, hMSCs (1 × 10^4^ cells) were seeded onto PCL- and PDA-coated PCL fibers and quantified by CCK–8 assay at three, seven and 14 days of culture. For each experimental time, samples were incubated with fresh medium containing 10% of CCK–8 reagent for four hours at 37 °C. After that time, the supernatant was removed and placed into 96–well plate to measure absorbance in a spectrophotometer (Victor X3 Multilabel Plate Reader PerkinElmer, Milan, Italy) at 450 nm.

## 4. Conclusions

Herein, the fabrication of fibrous platforms with electrically conductive surfaces was optimized by using electro fluid dynamic techniques to fabricate PDA-coated PCL fibers. Morphological analyses confirmed that PDA can be synthesized and electro fluid dynamically processed to form surface coatings with different topographic features that can influence the interaction in vitro with cells. Biocompatibility studies confirmed, at this preliminary stage, no cytotoxic response of hMSC to the PDA coating, which also promotes an increase in cell proliferation until 14 days, thus opening a new interesting perspective for the application of bio–conductive fibers in tissue engineering.

Future studies will be focused on the evaluation of electrical conductivity of the fiber surface in order to evaluate their contribution to in vitro cell response in 3D in vivo-like models.

## Figures and Tables

**Table 1:** Thermal decomposition temperature and melting heat of untreated and treated PCL fibers.

Sample	T_max_ (°C)	T_m_ (°C)
**PCL**	403.0 ± 0.3	59.4 ± 0.4
**PCL+NaOH**	403.7 ± 0.4	59.8 ± 0.3
**PCL+NaOH+PDA**	398.6 ± 0.5	60.1 ± 0.4
**PC** **L+PDA**	400.0 ± 0.4	60.9 ± 0.5

## References

[1] Guarino V., Cirillo V., Altobelli R., Ambrosio L. (2015). Polymer based platforms by electric field assisted techniques for tissue engineering and cancer therapy. Expert Rev. Med. Devices.

[2] Mijovic B., Trcin M.T., Agic A., Bujic E.Z.M., Spoljaric I., Kosec V. (2012). Study on cell adhesion detection onto biodegradable electrospun PCL scaffolds. J. Fiber Bioeng. Inform..

[3] Pires L.R., Guarino V., Oliveira M.J., Ribeiro C.C., Barbosa M.A., Ambrosio L., Pêgo A.P. (2016). Loading poly (trimethylene carbonate-co-ε-caprolactone) fibers with ibuprofen towards nerve regeneration. J. Tissue Eng. Regen. Med..

[4] Cirillo V., Guarino V., Alvarez–Perez M.A., Marrese M., Ambrosio L. (2014). Optimization of fully aligned bioactive electrospun fibers for “in vitro” nerve guidance. J. Mater. Sci. Mater. Med..

[5] Tan P.S., Teoh S.H. (2007). Effect of stiffness of polycaprolactone (PCL) membrane on cell proliferation. Mater. Sci. Eng. C.

[6] Qin X., Wu D. (2012). Effect of different solvents on poly(caprolactone) (PCL) electrospun nonwoven membranes. J. Therm. Anal. Calorim..

[7] Bosworth L.A., Hu W., Shi Y., Cartmell S.H. (2019). Enhancing biocompatibility without compromising material properties: An optimised NaOH treatment for electrospun polycaprolactone fibers. J. Nanomater..

[8] Guarino V., Cirillo V., Ambrosio L. (2016). Bicomponent electrospun scaffolds to design ECM tissue analogues. Expert Rev. Med. Devices.

[9] Liu Y., Ai K., Lu L. (2014). Polydopamine and its derivative materials: Synthesis and promising applications in energy, environmental, and biomedical fields. Chem. Rev..

[10] Khan Z., Shanker R., Um D., Jaiswal A., Ko H., Khan A., Jawaid M., Khan A.A.P., Asiri A.M. (2018). Bioinspired polydopamine and composites for biomedical applications. Electrically Conductive Polymers and Polymer Composites: From Synthesis to Biomedical Applications.

[11] Liebscher J. (2019). Chemistry of polydopamine—Scope, variation, and limitation. Eur. J. Organ. Chem..

[12] De Falco F., Guarino V., Gentile G., Cocca M., Ambrogi V., Ambrosio L., Avella M. (2019). Design of functional textile coatings via non conventional electrofluidodynamic processes. J. Colloid Interface Sci..

[13] Guarino V., Cirillo V., Taddei P., Alvarez–Perez M.A., Ambrosio L. (2011). Tuning size scale and cristalliniy of PCL electrospun membranes via solvent permittivity to adress hMSC response. Macromol. Biosci..

[14] Ryu J.H., Messersmith P.B., Lee H. (2018). Polydopamine surface chemistry: A decade of discovery. ACS Appl. Mater. Interfaces.

[15] Du W.W., Zou H., Tian M., Zhang L.Q., Wang W.C. (2012). Electrically conductive acrylonitrile–butadiene rubber elastomers prepared by dopamine-induced surface functionalization and metallization. Polym. Adv. Technol..

[16] Nieto C., Vega M.A., Marcelo G., Martín del Valle E.M. (2018). Polydopamine nanoparticles kill cancer cells. RSC Adv..

[17] Guaccio A., Guarino V., Alvarez–Perez M.A., Cirillo V., Netti P.A., Ambrosio L. (2011). Influence of electrospun fiber mesh size on hMSC oxygen metabolism in 3D collagen matrices: Experimental and theoretical evidences. Biotechnol. Bioeng..

